# Paternal activation of CB_2_ cannabinoid receptor impairs placental and embryonic growth via an epigenetic mechanism

**DOI:** 10.1038/s41598-019-53579-3

**Published:** 2019-11-19

**Authors:** Elisa Innocenzi, Emanuela De Domenico, Fabio Ciccarone, Michele Zampieri, Gabriele Rossi, Rosella Cicconi, Roberta Bernardini, Maurizio Mattei, Paola Grimaldi

**Affiliations:** 1 Department of Biomedicine and Prevention, University of Rome “Tor Vergata”, Rome, Italy; 2 IRCCS San Raffaele Pisana, Department of Human Sciences and Promotion of the Quality of Life, San Raffaele Roma Open University, Rome, Italy; 3 Department of Experimental Medicine, University of Rome “La Sapienza”, Rome, Italy; 4 Interdepartmental Service Centre–Station for Animal Technology (STA), University of Rome “Tor Vergata”, Rome, Italy; 5 Department of Biology, University of Rome “Tor Vergata”, Rome, Italy

**Keywords:** Embryogenesis, Risk factors

## Abstract

The cannabinoid receptor type 2 (CB_2_) is the peripheral receptor for cannabinoids, involved in the homeostatic control of several physiological functions. Male mitotic germ cells express a high level of CB_2_, whose activation promotes their differentiation in both *in vitro* and *in vivo* experiments, controlling the correct progression of spermatogenesis. However, it remains elusive if CB_2_ activation in spermatogonia could affect reproductive success in terms of fertility and healthy pregnancy outcomes. In this study, we explored the effects of male CB_2_ activation on sperm number and quality and its influence on next generation health. We show that exposure of male mice to JWH-133, a selective CB_2_ agonist, decreased sperm count, impaired placental development and reduced offspring growth. These defects were associated with altered DNA methylation/hydroxymethylation levels at imprinted genes in sperm and conserved in placenta. Our findings reveal that paternal selective activation of CB_2_ alters the sperm epigenome and compromises offspring growth. This study demonstrates, for the first time, a new role of CB_2_ signaling in male gametes in causing epigenetic alterations that can be transmitted to the next generation by sperm, highlighting potential risks induced by recreational cannabinoid exposure.

## Introduction

*Cannabis sativa* (Marijuana) is the drug most commonly used by young men and women and its usage is rising with its legalization. The main psychoactive constituent of cannabis, Δ9- tetrahydrocannabinol (THC) binds to and activates both cannabinoid receptors CB_1_ and CB_2_. CB_1_ is the most abundant G protein-coupled receptor expressed in the brain, while CB_2_ is mainly expressed in immune cells^1,2^. Cannabinoid receptors, together with their endogenous ligands and all the enzymes involved in endocannabinoids biosynthesis and degradation, form the endocannabinoid system (ECS)^3^. ECS is deeply involved in the regulation of male and female reproduction^4–6^. Interference with the delicate balance of the ECS in germ cells, by the use of exogenous cannabinoids, has an adverse effect on reproduction. The two main cannabinoid receptors, CB_1_ and CB_2_, are both involved in male reproductive biology and in the testis they have distinct expression and roles. CB_1_ is mainly expressed by Leydig cells and mature sperm and its activation negatively affects sperm functions by inhibiting motility, capacitation and acrosome reaction^7,8^. In absence of CB_1_ signaling, sperms acquire motility precociously, suggesting a physiological inhibitory regulation of endocannabinoids on their motility during the transition into the epididymis^9^. CB_2_ is expressed by Sertoli cells and, at a higher level, by spermatogonia and its activation promotes germ cell meiotic entry both *in vitro* and *in vivo*, regulating the correct progression of spermatogenesis^5^. Indeed, chronic administration of JWH-133, a potent and selective CB_2_ agonist, to young male mice causes an acceleration of spermatogenesis, while treatment with specific CB_2_ antagonist has the opposite effect, slowing down the process^10^. Nevertheless, so far, no information has been reported on the effects of male exposure to JWH-133 on sperm number, morphology and function as well as on the potential impact on the offspring. This last outcome is of particular interest for the possible transmission by sperm of epigenetic alterations that could interfere and influence the health and development of future generations. Epigenetic alterations such as DNA methylation, histone modifications and/or non-coding RNAs, can occur during spermatogenesis following drug exposure. A number of human and animal studies have begun to reveal the long-term impact of cannabis and cannabinoid exposure on the neurodevelopment and behavior of the next generation, outlining the aberrant epigenetic modifications in brain and in the periphery linked to cannabis exposure^11,12^. Very recently Murphy *et al*. reported altered DNA methylation in sperm from humans and rats after cannabis or tetrahydrocannabinol (THC) exposure^13^. Here we investigated the effects of chronic exposure of young male mice to JWH-133 on reproductive success in terms of sperm quality, fertility and healthy pregnancy outcomes. We show that paternal exposure to JWH-133 reduced sperm count and caused defects in placental and embryonic development. These effects were associated with altered DNA methylation and hydroxymethylation at specific imprinted genes in sperm and in placenta. Our results underline the urgent need for studies evaluating the potential risks of cannabis/cannabinoid usage for offspring development and health.

## Results

### Chronic administration of JWH-133 reduces sperm count

**Figure 1:**
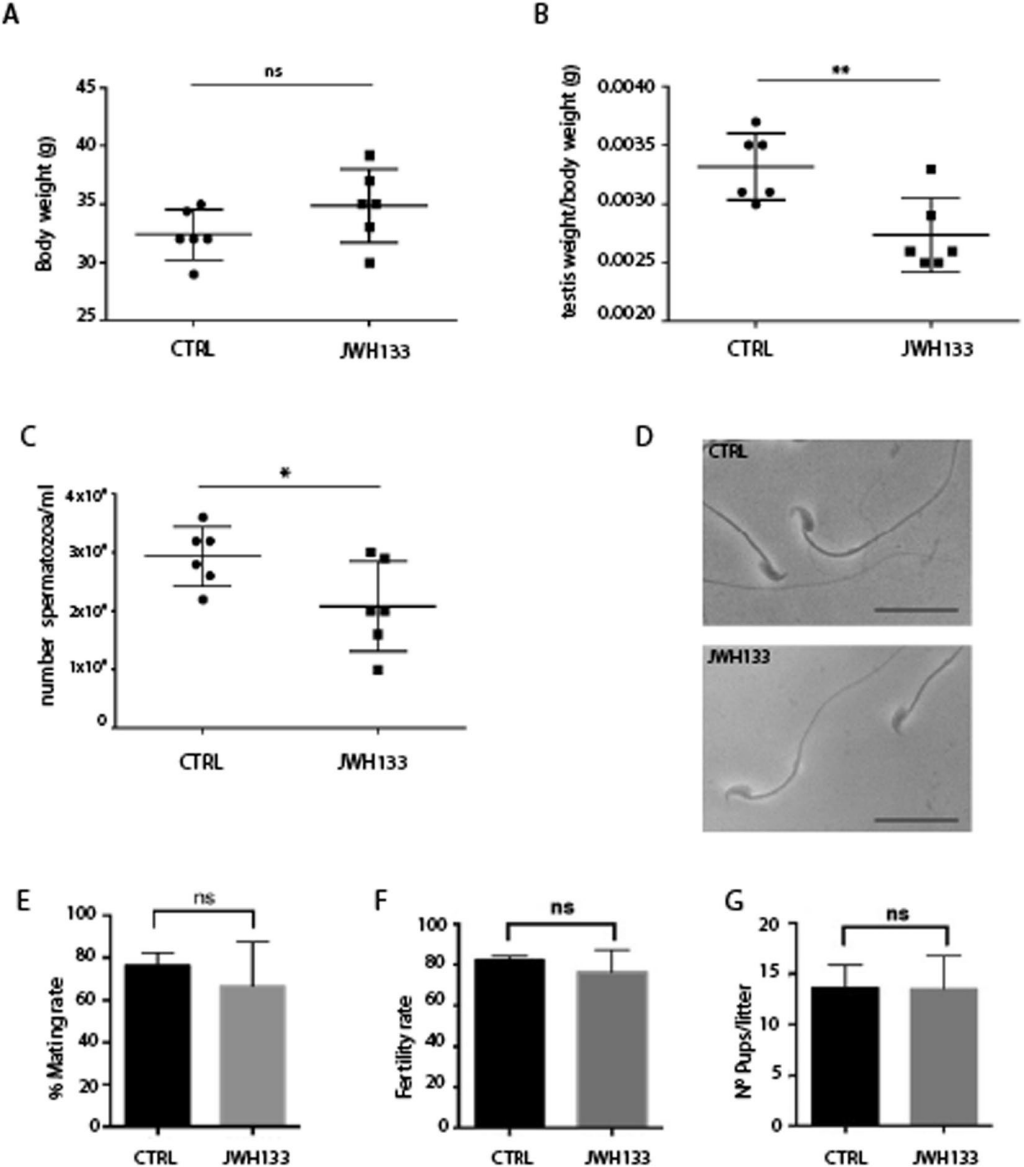
JWH-133 exposed males have a reduced sperm count. CD-1 male mice at P7 were chronically injected with JWH-133 (1.5 mg·kg^−1^) for 5 weeks as reported in Methods. Analysis was performed for (**A**) Body weight (34.87 ± 3.18 g in JWH-133 vs 32.40 ± 2.14 g in CTRL); (**B**) testis weight/body weight (2.73 ± 0.31 mg in JWH-133 vs 3.32 ± 0.29 mg in CTRL; (**C**) number of spermatozoa/ml; (**D**) sperm shape and motility (Scale bar: 25 µm). JWH-133 treated mice (n = 6) have a significantly decreased testis weight and sperm number compared with control untreated group (n = 6), while their shape and motility appear normal. Control (n = 6) and JWH-133 treated mice (n = 6) were crossed with sexually mature female mice never exposed to the drug (single male pared with single female, n = 3 total females) for one month and mating rate (**E**), fertility rate (**F**) and the number of pups/litter (**G**) were analyzed. *P < 0.05; **P < 0.01. Error bars represent SD.

Young male mice at P7 were injected with JWH-133 (1.5 mg/kg)^10,14,15^ or vehicle (see methods) and, at the end of 5 weeks, they were weighed and sacrificed. No differences between control and treated mice in body weight were detected at adulthood when they reached sexual maturity (Fig. 1A), while a significant reduction in testis weight of treated males was observed (Fig. 1B). To understand if the observed reduction in testicular volume was associated with a reduction in the number of spermatozoa, cauda epididymis were collected and needled to allow spermatozoa to “swim-out”. As indicated in Fig. 1C a significant reduction in the number of sperm from treated mice (2.08 ± 0.76 × 10^6^/ml) with respect to control mice (2.93 ± 0.50 × 10^6^/ml) was observed. However, sperms from treated mice were normally developed, as they did not show shape abnormalities nor altered motility (Fig. 1D).

To test the effect of JWH-133 treatment on male mating and fertility, adult JWH-133 treated male mice were crossed with untreated sexually mature CD-1 females. Vaginal plugs were evaluated every morning to determine if mating had occurred and each mated mouse was isolated in a separate cage. The mating rate was calculated as mean of the number of plugs occurring every morning for a week, while fertility rate was evaluated as the number of certainly pregnant females over the total number of plugged females. JWH-133 treatment of male mice did not cause significant alterations in mating rate (69.0 ± 18.66% in JWH-133 vs 79.50 ± 6.54% in control, Fig. 1E), fertility rate (76.2 ± 11% in JWH-133 vs 82 ± 2.01% in control, Fig. 1F) and litter size (13.65 ± 2.2% in JWH-133 vs 13.5 ± 3.3% in control, Fig. 1G). We conclude that chronic treatment of young male mice for 5 weeks with JWH-133 reduced sperm count without impacting fertility rate and litter size.

### Paternal exposure to JWH-133 impairs offspring growth

**Figure 2:**
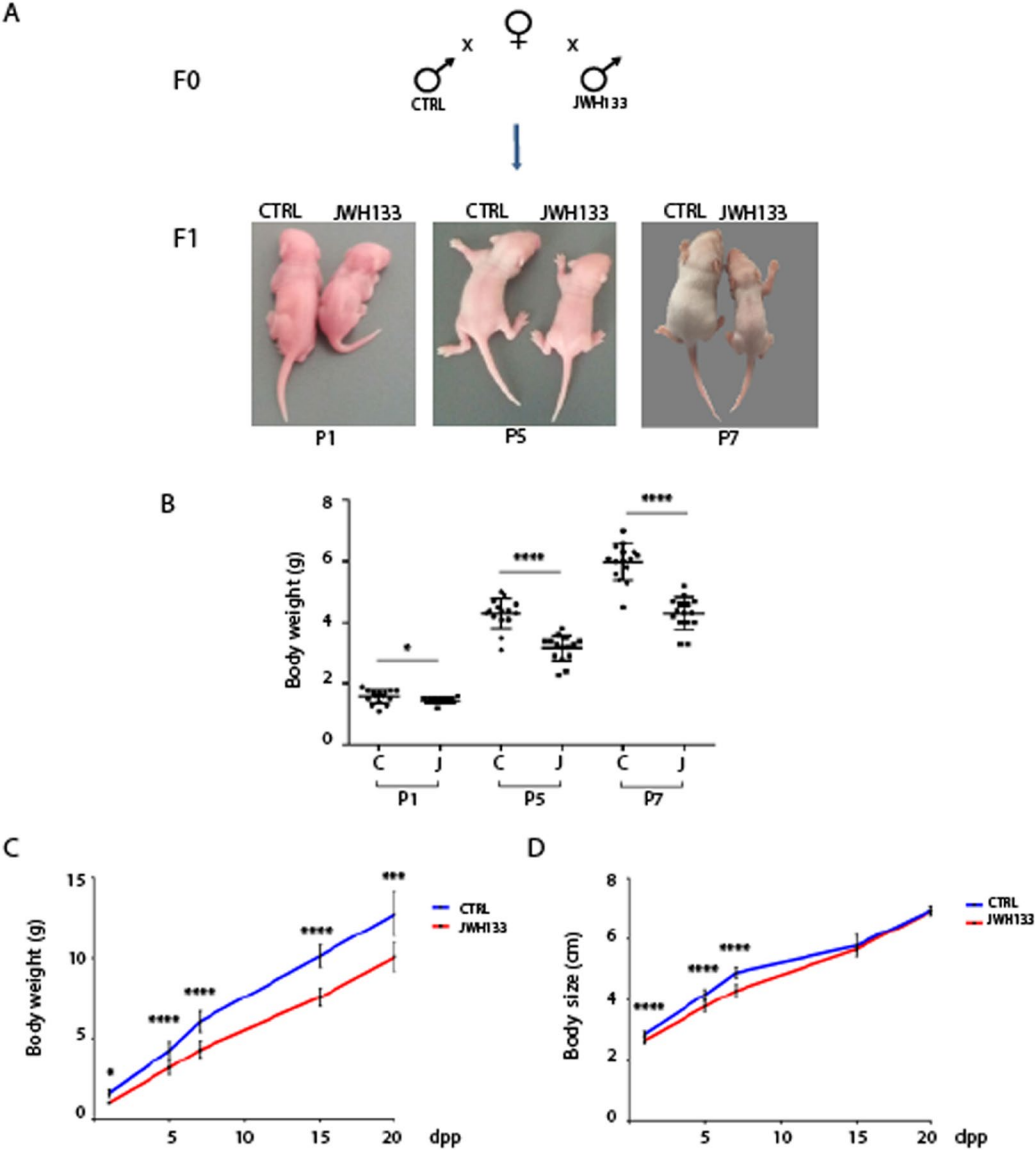
Paternal exposure to JWH-133 causes growth defects in the offspring. CTRL and JWH-133 treated males were crossed with mature untreated females and F1 offspring was analyzed. (**A**) Representative images of F1 pups at P1, P5 and P7 from control and JWH-133 exposed males. (**B**) Scatter plot showing results of n = 15 pups at P1, P5 and P7 randomly selected from n = 3 litters from each group. (**C**) Body weight and (**D**) body size of pups from control and JWH-133 exposed fathers, from birth up to 20 dpp (see also Table 1). Results are the mean from n = 80 pups from JWH-133 father and n = 80 pups from control father, derived from three different matings. *P < 0.05;***P < 0.001; ****P < 0.0001. Error bars represent SD.

In order to test the capacity of sperm from JWH-133-exposed males to generate healthy offspring, adult males were bred with mature CD-1 females never exposed to the drug and their F1 litters were examined at E13.5, E18.5, P1, P5, P7, P15, P20 and adult age. Although the pregnancy rate of females mated with JWH-133 treated males did not change compared with females mated with control males, we found that JWH-133 sired had lower birth weight and size (Table 1). Representative images of control and JWH-133 sired pups at P1, P5 and P7 are shown in Fig. 2A and their difference in body weight recorded at these ages is shown by scatter plot in Fig. 2B. Difference in body weight was maintained until adult age (Fig. 2C), while body size was recovered by P15, reaching the same parameters of control pups (Fig. 2D). Frequency of these birth abnormalities was observed in every litter and included males and females. Some organs from control and treated mice were weighed at P7, time at  which difference between control and JWH-133 sired pups was clearly evident. As shown in Fig. S1A each organs’ weight of pups from treated mice was decreased, according to their reduced body weight. Consequently the ratio between organ weight and body weight did not show significant changes for each organ (Fig. S1B) indicating that treatment of fathers with JWH-133 impacted the overall development of the embryos and did not alter specific organ development. Furthermore, in order to exclude the presence of alterations in reproductive organs of F1 pups, male and female gonads of pups from control and JWH-133 exposed fathers were morphologically analyzed. Representative images of histological sections of testis and ovary at P7 showed no apparent abnormalities (Fig. S1C,D), indicating that male and female germ cells were present and normally developed. In addition, to investigate fertility of F1 mice from JWH-133 treated father, males were crossed with untreated females for four consecutive weeks. Accordingly with the normal development of the gonad, they showed no changes in mating, fertility rate and litter size respect with control mice (Fig. S1E), suggesting that fathers’ exposure to JWH-133 did not interfere with the reproductive function of F1 progeny. However, further studies will be required to understand if sperm epigenetic modifications of the exposed father are inherited by F1 males and transmitted transgenerationally.

**Table 1:** Weight and size of F1 pups from control and JWH-133 fathers at different postnatal age.

		CTRL	JWH-133
Weight	*1dpp*	1.58 ± 0.23	1.43 ± 0.09
	*5dpp*	4.27 ± 0.54	3.16 ± 0.42
	*7dpp*	6.05 ± 0.65	4.30 ± 0.53
	*15dpp*	10.15 ± 0.68	7.58 ± 0.59
	*20dpp*	12.80 ± 1.39	10.09 ± 0.88
Length	*1dpp*	2.85 ± 0.09	2.65 ± 0.11
	*5dpp*	4.17 ± 0.18	3.77 ± 0.18
	*7dpp*	4.89 ± 0.18	4.30 ± 0.21
	*15dpp*	5.78 ± 0.35	5.67 ± 0.09
	*20dpp*	6.98 ± 0.12	6.96 ± 0.15

### Paternal exposure to JWH-133 causes embryonic and placental size defects

**Figure 3:**
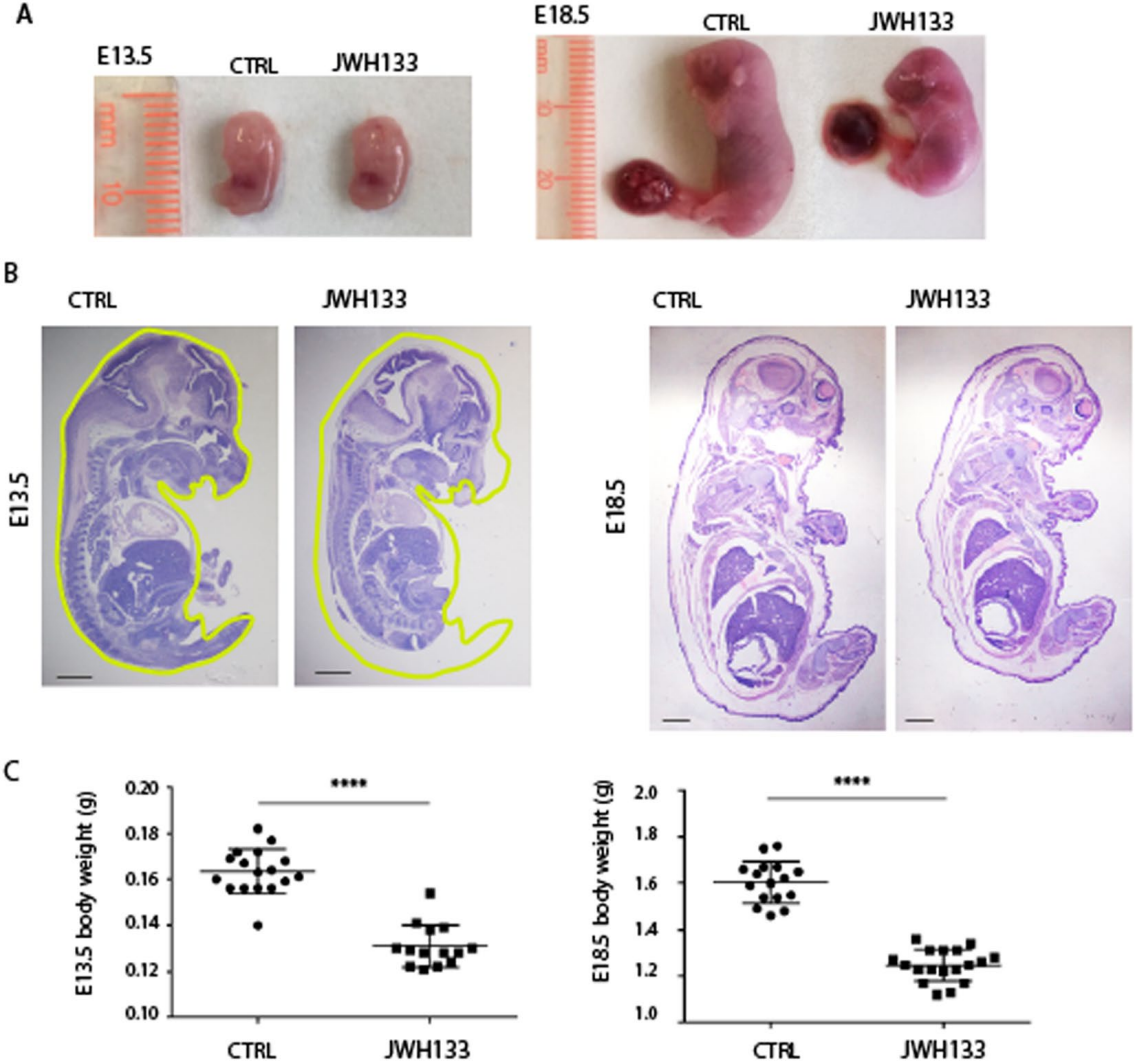
Growth defects in embryo sired from JWH-133 exposed father. (**A**) Representative images of E13.5 and E18.5 embryos from JWH-133 exposed and control (CTRL) fathers. (**B**) Histological sections (H&E staining) of E13.5 and E18.5 embryos from JWH-133 exposed and control (CTRL) fathers, displaying a reduced embryo size without developmental abnormalities. The outline of the CTRL embryo is traced in yellow and transferred in JWH-133 embryo to illustrate and compare the overall size between the two embryos. (**C**) Embryo body weight at E13.5 (130.0 ± 9.14 mg in JWH-133 vs 163.4 ± 9.84 mg in CTRL) and E18.5 (1.25 ± 0.07 g in JWH-133 vs 1.60 ± 0.09 g in CTRL). Scatter plots show the results of n = 15–18 embryos randomly selected from n = 3 females from each group. ****P < 0.0001. Scale bar: 1 mm in B.

**Figure 4:**
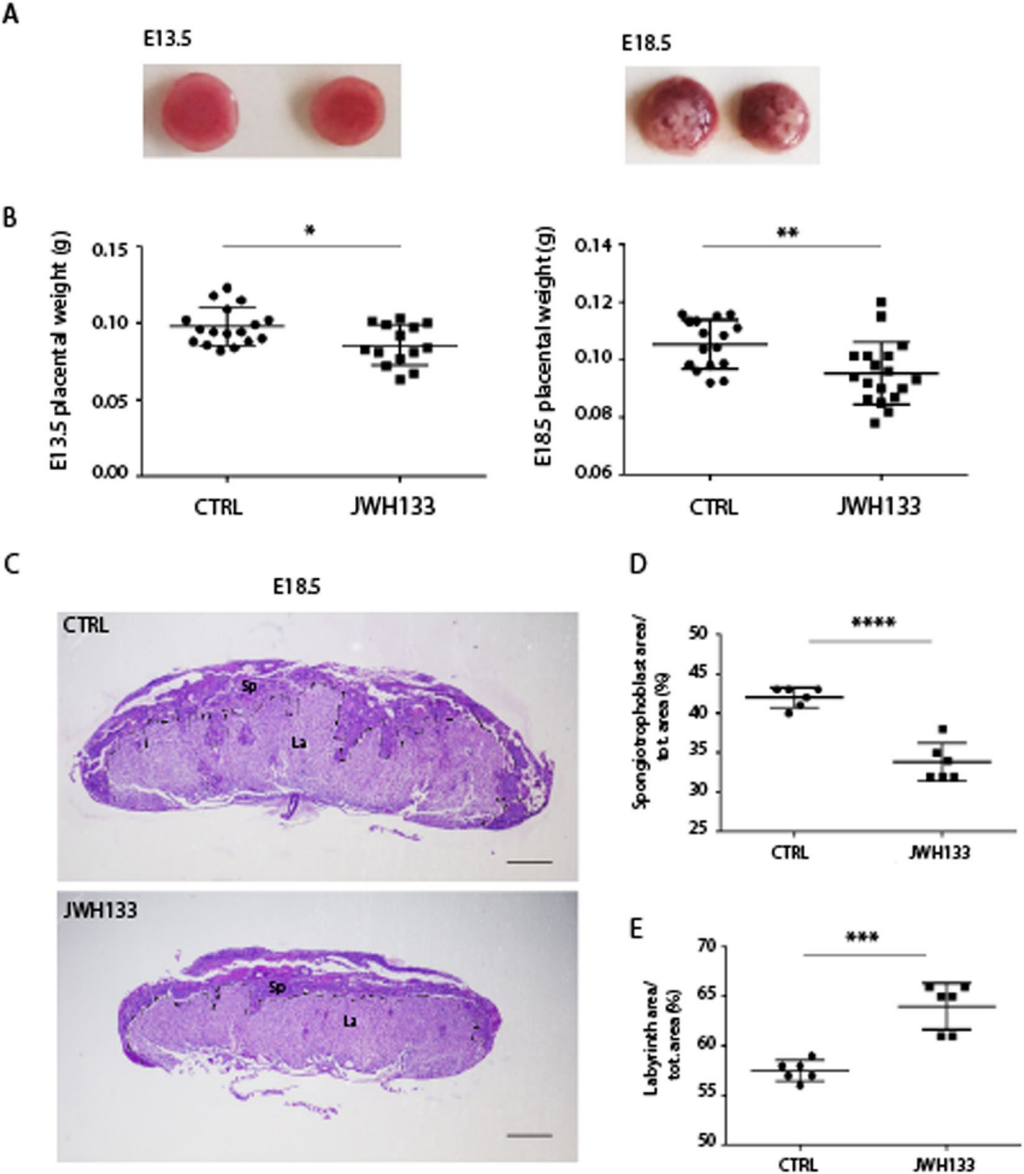
Placental abnormalities in pregnancies sired by a JWH-133 treated father. (**A**) Representative images of E13.5 and E18.5 placentas from JWH-133 exposed and control (CTRL) fathers. (**B**) Scatter plots of placental weight at E13.5 and E18.5 from JWH-133 exposed and control (CTRL) fathers showing a significant reduction in those from treated males (E13.5: 85.71 ± 13.20 mg in JWH-133 vs 97.82 ± 12.22 mg in CTRL; E18.5: 95.22 ± 10.87 mg in JWH-133 vs 105.3 ± 8.48 mg in CTRL). Scatter plots show the results of n = 15–18 placentas randomly selected from n = 3 females from each group. *P < 0.05; **P < 0.01. (**C**) Histology of E18.5 placenta cross-sections from fetus sired by CTRL or by JWH-133 father stained with PAS. The spongiotrophoblast (Sp) and the labyrinth (La) are outlined manually showing a thinner Sp layer in the placenta from JWH-133 treated male. (**D**) Scatter plot of the ratio of Sp/total area in placentas from CTRL and JWH-133 male (33.83% ± 2.401 in JWH-133 vs 42.00% ± 1.265 in CTRL) (**E**) Scatter plot of the ratio of La/total area in placenta from CTRL and JWH-133 male (64.00% ± 2.366 in JWH-133 vs 57.50% ± 1.049 in CTRL). The areas were measured by using five sections for each placenta. Scatter plots show the results of n = 6 placentas randomly selected from each group. ***P < 0.001; ****P < 0.0001. Scale bar: 1 mm in C.

**Figure 5:**
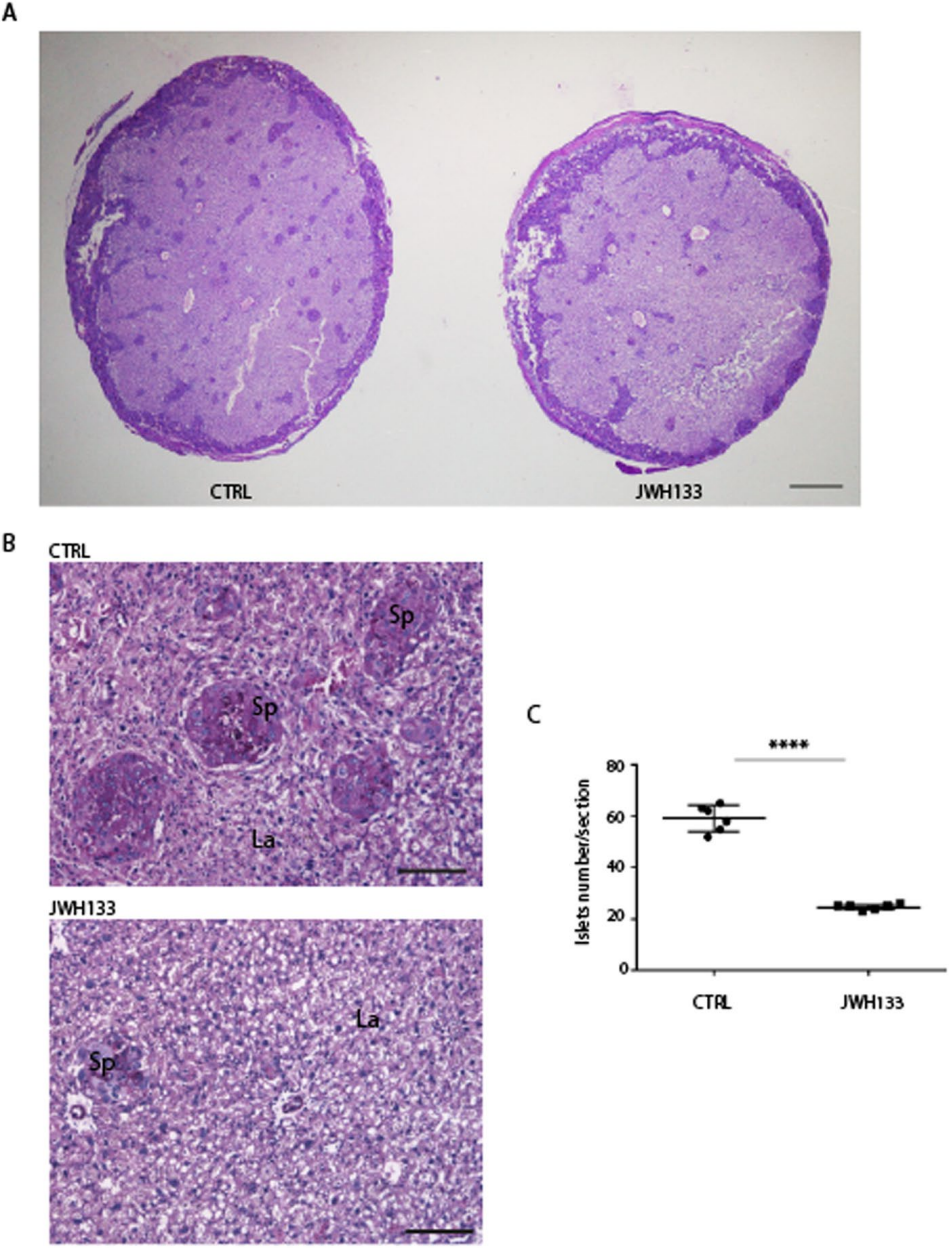
Reduced number of spongiotrophoblast islets in E18.5 placentas from JWH-133 father. (**A**) PAS staining of placenta cross-sections from CTRL and JWH-133 fathers. JWH-133 derived placenta is smaller than CTRL placenta. Glycogen cells are stained deep purple by PAS. A reduced number of glycogen cell islets within the labyrinth layer was observed in the placenta from JWH-133 father. This is consistent with the presence of a thinner and less invasive spongiotrophoblast layer. Scale bar: 1 mm. (**B**) Magnification view of the spongiotrophoblast islets. Scale bar: 100 µm. (**C**) Scatter plot of the number of islets/section showing the reduced number of islets in placenta from JWH-133 father (24.67 ± 1.03 in JWH-133 vs 59.17 ± 5.04 in CTRL). The number was counted by using five cross-sections for each placenta (n = 5) for each group. Error bars show SD. ****P < 0.0001.

In order to investigate if low weight and small size at birth of pups from JWH-133 fathers were the result of embryonic developmental defects, E13.5 and E18.5 embryos were analyzed. We observed that paternal exposure to JWH-133 caused an evident decrease in embryo size and weight with respect to embryos from control fathers (Fig. 3A,C). However, histological examination of E13.5 and E18.5 whole embryos showed no organ-specific abnormalities in fetuses sired by JWH-133 males (Fig. 3B), suggesting that drug administration to the father negatively affected overall embryo growth. Defects in embryo growth are often associated with abnormalities in placental development. Indeed the placenta plays a critical role in the growth and survival of the fetus by promoting nutrient transfer from mother to fetus and changes in placental glycogen deposition are a common feature of pregnancy complications, particularly those associated with altered fetal growth^16^. Mouse placenta can be divided into a maternal decidual component and two fetal layers, the labyrinth zone (La) and the spongiotrophoblast layer (Sp). To determine if paternal JWH-133 exposure affected placental development, placentas were collected at E13.5 and E18.5 (Fig. 4A) and morphologically analyzed. The placental weight of fetuses sired from JWH-133 males was significantly lower with respect to the placentas of fetuses from control mice (Fig. 4B). Histological examination of sections of placenta at late gestation (E18.5) showed that those derived from JWH-133 mice had a significantly reduced total area (Fig. 4C). Interestingly, after PAS staining that allows to better visualize the spongiotrophoblast containing glycogen cells, measurement of the area of each layer showed that the percentage of spongiotrophoblast layer/total area was significantly smaller in JWH-133 placentas (n = 6) than in control placentas (n = 6) (33.83% ± 2.40 in JWH-133 vs. 42.0% ± 1.26 in control, P < 0.0001, Fig. 4D), while the percentage of labyrinth zone/total area was increased (64.0% ± 2.37 in JWH-133 vs. 57.5% ± 1.05 in control, P < 0.001, Fig. 4E). Moreover, a strongly reduced number of islets of the spongiotrophoblast layer in the labyrinth zone was found in placentas from JWH-133 mice with respect to control placentas (Fig. 5A,B). The number of independent islets was quantified in the placentas of two groups, demonstrating that paternal exposure to cannabinoid JWH-133 caused them a strong reduction (24.67 ± 1.03 in JWH-133 vs. 59.17 ± 5.04 in control, P < 0.0001, Fig. 5C), indicating the presence of a thinner and less invasive spongiotrophoblast layer.

### Altered DNA methylation in sperm of JWH-133 exposed males

**Figure 6:**
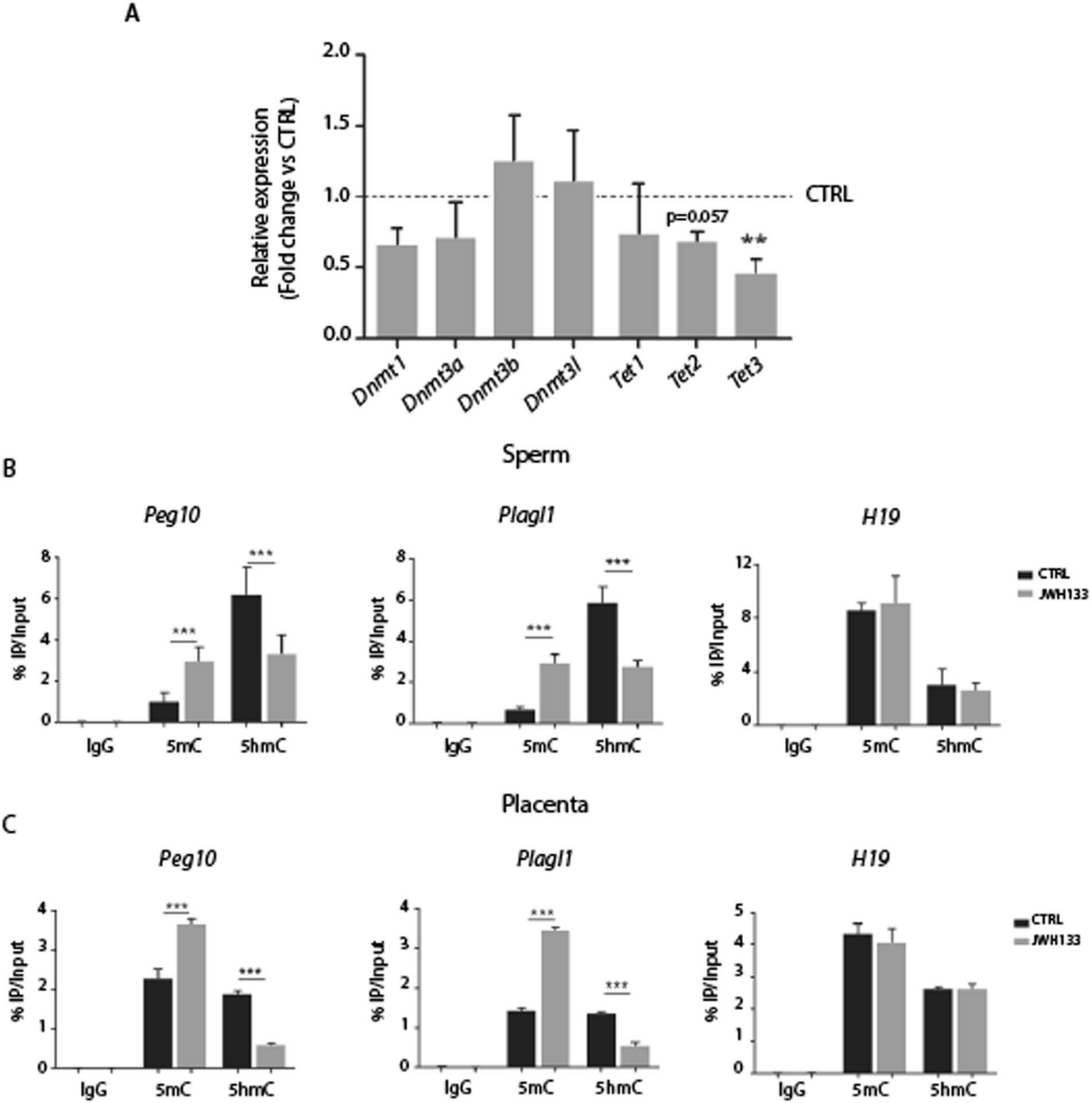
Altered DNA methylation and hydroxymethylation levels in sperm from JWH-133 exposed males and in the placenta of their progeny. (**A)** Histogram reporting gene expression analysis of the DNA methyltransferases (*Dnmts*) and *Tet* hydroxylase performed by RT-qPCR on sperm RNA from JWH-133 exposed males (n = 6) with respect to controls (n = 6) that is set as 1 (*Dnmt1*: 1 ± 0.19; *Dnmt3a*: 1 ± 0.27; *Dnmt3b*: 1 ± 0.15; *Dnmt3l*: 1 ± 0.52; *Tet1*: 1 ± 0.41; *Tet2*: 1 ± 0.11; *Tet3*: 1 ± 0.09). b-Actin is used as reference gene. (**B**) MeDIP/hMeDIP analysis performed on pooled DNA obtained from sperm of six JWH-133 exposed males and six controls showing the enrichment of 5mC and 5hmC on imprinted genes. (**C**) MeDIP/hMeDIP analysis performed on pooled DNA obtained from placentas (n = 6) of three females crossed with JWH-133 exposed or control males. IgGs are used as controls and data obtained are shown as percentage of input DNA. Selected imprinted genes are *Peg10* and *Plagl1*, paternally expressed and *H19*, maternally expressed. Results are reported as mean ± SD and statistical significance was obtained by Student’s t-Test; (**p < 0.01;***p < 0.001).

Previous studies have established a critical function of some imprinted genes in embryonic and placental development^17,18^. Imprinted genes rely on DNA methylation to silence and activate alleles in a parent-of-origin specific manner, ensuring the reciprocal expression of specific genes. In mammals, *de novo* DNA methylation is established by DNMT3A and DNMT3B and is then maintained by DNMT1 during cell division^19^. Instead, TET proteins, including TET1, TET2, and TET3, are crucial regulators of active DNA demethylation and catalyze the oxidation of 5-methylcytosine (5mC) to 5-hydroxymethylcytosine (5hmC)^20^. To understand the molecular events responsible for the phenotypic alterations observed in placentas and embryos from JWH-133 males, we analyzed the expression level of key genes responsible for DNA methylation (*Dnmt1, Dnmt3a, Dnmt3b, Dnmt3l*) and DNA hydroxymethylation (*Tet1-3*), in sperm from treated and untreated mice. We found that *Tet3* gene expression was significantly decreased (p < 0.01) in sperm of exposed males as well as the expression of *Tet1* and *Tet2,* although not significantly. On the other hand, no changes were detected for *Dnmts* expression levels (Fig. 6A). In order to investigate whether the observed reduction of *Tet* genes expression was functionally correlated to a change in DNA methylation and hydroxymethylation levels of imprinted genes involved in placental and embryonic growth/development, we focused our analysis on *H19*, *Peg10* and *Plagl1 genes*. *H19* is a maternally expressed imprinted gene that is hypermethylated in sperm and functions as a trans regulator of other imprinted genes during embryo growth^21,22^*, Peg10* is a paternally expressed gene essential for the formation of the placenta in humans and mice^23^ and *Plagl1* (also known as *ZAC*, *LOT1* and *Zac1* in mouse) is a paternally expressed gene and key regulator of a network of other imprinted genes, involved in embryonic growth and development^24^. DNA methylation/hydroxymethylation level of these target genes was assessed by DNA immunoprecipitation with anti-5mC and anti-5hmC antibodies, followed by real-time PCR (Fig. 6B). Interestingly, we found a significant enrichment of 5mC at *Peg10* and *Plagl1* genes in sperm of JWH-133 males concomitantly with a decrease in 5hmC, while no changes in DNA methylation/hydroxymethylation were observed for *H19* gene which is maternally expressed (Fig. 6B). Next, we investigated the methylation levels of these genes in E13.5 placentas derived from control and treated males. We found that placentas from JWH-133 males showed alterations in 5mC and 5hmC levels at *Peg10* and *Plagl1* genes similar to those observed in sperm of JWH-133 treated males (Fig. 6C). In conclusion, our results indicate that paternal exposure to CB_2_ agonist JWH-133 causes alterations in the sperm epigenome, which are then transferred to embryonic tissues and may account for the observed growth defects.

### Chronic exposure to JWH-133 affects the immune system

**Figure 7:**
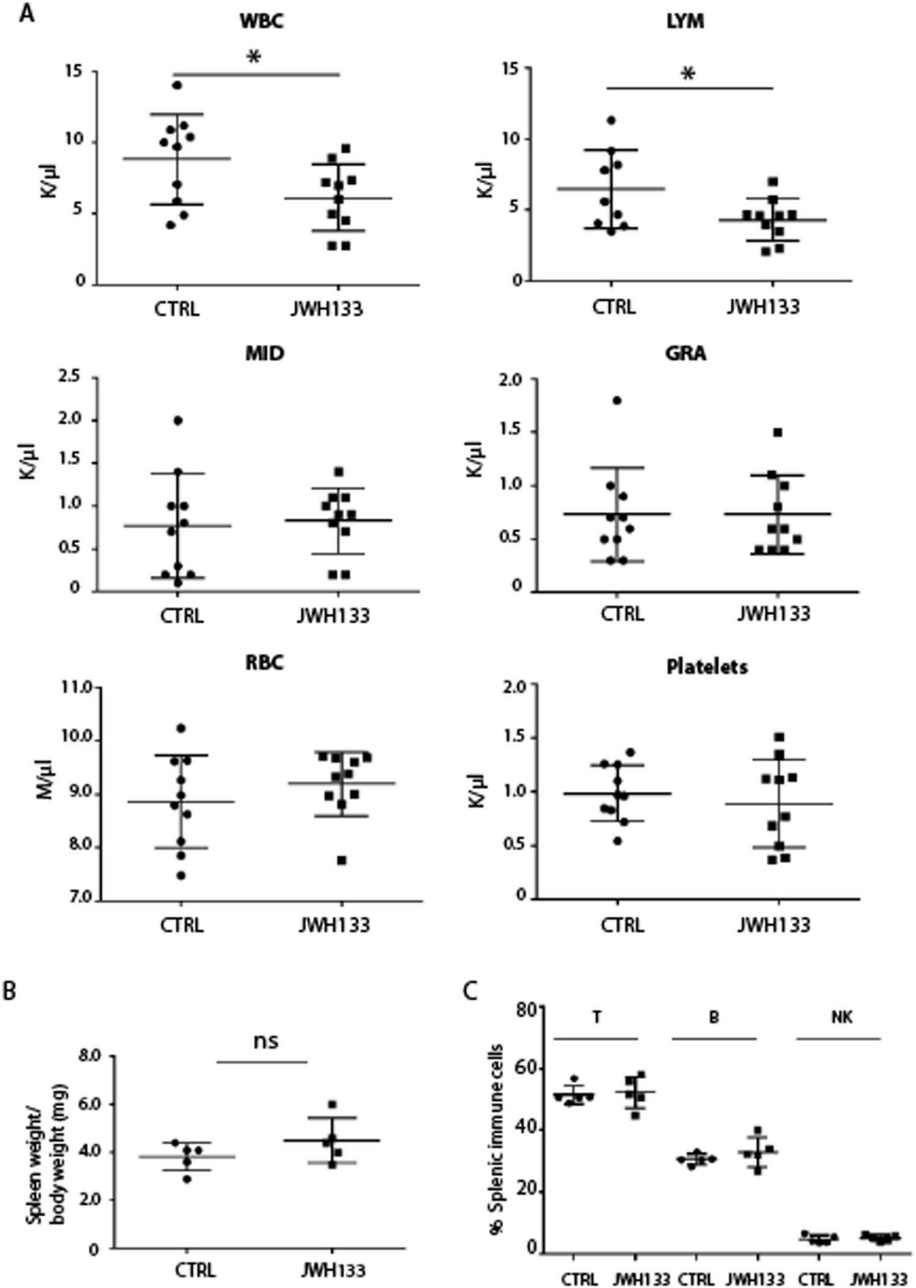
Altered immune system in JWH-133 exposed male. (**A**) Scatter plot reporting the number of circulating white blood cells (WBC), lymphocyte (LYM), mid-sized cells (MID) and granulocyte (GRA), red blood cells (RBC) and platelets in JWH-133 exposed male mice (n = 10) and in control mice (n = 10) showing a significant reduction in WBC and lymphocytes in drug exposed male (*P < 0.05). (**B**) Scatter plot showing the spleen weight/body weight in CTRL and JWH-133 exposed males (n = 5 for each group). (**C**) Scatter plot showing the percentage of splenic lymphocyte populations obtained by FACS analysis. The antibody used for the detection of T-lymphocytes was anti-CD3-PE-Cy5; for detection of B-lymphocytes was anti B-220-FITC; for detection of Natural Killer cells (NK) was anti-NK-PE.

It is well established that CB_2_ is abundantly expressed in cells of the immune system^2,25^ and that selective activation of this receptor down-regulates immune response. Therefore, CB_2_-selective agonists have been demonstrated to play an anti-inflammatory and immunosuppressive role in mice models. In order to verify the health status of mice chronically exposed to JWH-133, we evaluated hematological parameters on blood samples collected from the orbital sinus through a hemocytometer. We report that drug treatment caused a significant decrease in the number of white blood cells (WBC, 6.12 ± 2.34 K/µl in JWH-133 vs 8.83 ± 3.16 K/µl in controls) and circulating lymphocytes (LYM, 4.32 ± 1.47 K/µl in JWH-133 vs 6.48 ± 2.75 K/µl in controls), while no differences were detected in mid-size cells (MID, 0.83 ± 0.38 K/µl in JWH-133 vs 0.77 ± 0.61 K/µl in controls), granulocytes (GRA, 0.73 ± 0.37 K/µl in JWH-133 vs 0.73 ± 0.44 K/µl in controls) as well as in red blood cells (RBC, 9.2 ± 0.60 M/µl in JWH-133 vs 8.86 ± 0.86 M/µl in controls) and platelets (892.0 ± 405.9 K/µl in JWH-133 vs 985.8 ± 261.9 in controls) (Fig. 7A). Spleen size and weight were similar between control and treated mice (Fig. 7B) as well as the number of T, B and NK lymphocytes analyzed by FACS (Fig. 7C). All together these results indicate that *in vivo* administration of JWH-133, reduced the number of WBC and circulating lymphocytes without affecting splenic lymphocyte populations.

## Discussion

This study aimed at evaluating the involvement of CB_2_ cannabinoid receptor in male reproductive performance. We previously reported that in the testis CB_2_ is expressed at a high level by spermatogonia and its *in vivo* activation, by using JWH-133, a synthetic CB_2_‐selective cannabinoid agonist, caused alteration of the temporal progression of spermatogenesis^5,10^. Here we report evidence that chronic exposure of young male mice to JWH-133 altered sperm count and epigenome affecting offspring growth. JWH-133 drug has been recently indicated as one of the best recommended CB_2_ selective agonists to study the role of CB_2_ in biological and disease processes^26^. Indeed, JWH-133 has a high potency (inhibitor constant = 3.4 nM) and selectivity (200‐fold more binding affinity over CB_1_) to CB_2_ and it has been suggested very suitable for preclinical studies^26,27^. Here we show that overactivation of CB_2_ signaling in young males affected spermatogenesis by decreasing sperm count of about 30%, without altering their motility, a function instead correlated to CB_1_ activation^7,9^. Production of a correct sperm count is under strict control of testosterone and alteration in its level impairs fertility^28^. CB_1_ has been indicated as an important regulator of testosterone production in testis since its activation inhibits basal testosterone secretion *in vivo* and *in vitro*. Indeed CB_1_ is expressed in hypothalamus, pituitary gland and Leydig cells, while CB_2_ receptor has never been identified there^29^. Thus, we are confident that our results on the decrease of sperm count, induced by activation of CB_2_ and associated to unchanged mating and fertility, is not caused by altered testosterone level. Instead we speculate that this reduction in sperm count is determined by an apoptotic effect of the drug on CB_2_ expressing germ cells. Accordingly, we previously observed a similar effect in fetal oocytes, in which activation of CB_2_ induced their differentiation but concomitantly determined apoptotic cell death^30^. Although chronic exposure to JWH-133 caused a significant decrease in sperm count, mating and fertility were not impaired and no reduction of litter size nor frequency was observed. Additional studies will be needed in order to elucidate the molecular mechanisms involved in germ cell apoptosis induced by CB_2_ overactivation.

To test the function of sperm from JWH-133 exposed mice, we evaluated its capacity to generate healthy offspring after crossing with unexposed females. We found that pups, sired from JWH-133 male mice, were smaller and lighter at birth than those sired from control untreated mice, indicating that paternal cannabinoid administration altered offspring growth. Accordingly, we observed that growth defects were already evident in E13.5 and E18.5 embryos and were maintained after birth up to one month, when pups begin feeding themselves and become independent from maternal lactation. Several causes can affect fetal growth amongst which placental defects are the most common. Abnormal placental development underlies a wide range of complications during pregnancy, including intrauterine growth restriction, preeclampsia and miscarriage^31–33^. Analysis of placentas from females fertilized by JWH-133 exposed fathers, showed a reduction in total weight by about 10–15%, with a thinner spongiotrophoblast layer (Sp) with respect to placentas from females fertilized by control fathers.

Placental and embryonic development depends on information transmitted from the gametes and epigenetic modifications are important determinants for gamete quality and offspring development. DNA methylation is one of the most studied epigenetic modifications involved in gene silencing. The steady-state level of methylation of a gene is determined by a balance between the actions of DNMT and TET enzymes. While DNMT enzymes establish DNA methylation patterns^19^, *TET* proteins play an important role in active DNA demethylation, by catalyzing the oxidation of 5-methylcytosine (5mC) to 5-hydroxymethylcytosine (5hmC)^20^. We found an evident decrease in the expression level of all *Tet* genes in sperm from JWH-133 exposed males, especially significant for *Tet3*, while the expression of *Dnmt*s was not significantly modified. As *TET* enzymes promote DNA demethylation, their reduced expression could be compatible with an increased DNA methylation. We focused our attention on imprinted genes because (i) they are expressed in a parent-of-origin specific manner through epigenetic processes occurring during gametogenesis and maintained in the developing embryo^34^; (ii) several paternally expressed imprinted genes are crucial for placental development and fetal growth: *DIO3*, *DLK1*, *HYMAI*, *IGF2*, *MAGEL2*, *MEST*, *PEG10*, *PEG3*, *PLAGL1*, *SFRP2*^35,36^. Among these genes, we selected *Peg10* and *Plagl1* for some considerations. *Peg10*, since it is a highly conserved gene among mammalian species and *Plagl1* since it is considered a master regulator of growth and development of the fetus/placenta. *Peg10* is a conserved retrotransposon-derived gene that is methylated in the female germline and its deletion causing severe defects in placenta formation and embryonic growth^23^. *Plagl1* encodes a zinc finger transcription factor and has been shown to upregulate a cluster of imprinted genes (H19, IGF2, CDKN1C) in mice and its loss results in severe embryo growth restriction^24^. We showed a reduced DNA hydroxymethylation and an increased DNA methylation at these paternally expressed genes in sperm from JWH-133-exposed males. Notably, the maternally expressed H19 gene was unaffected by JWH-133 treatment. Interestingly, DNA hypermethylation of *Peg10* and *Plagl1* genes in sperm was found conserved in placentas from JWH-133 exposed fathers. These results indicate that JWH-133-dependent defects in the sperm epigenome can be inherited by embryonic tissues and may be responsible for the observed growth impairment, also via downregulation of *Peg10* and *Plagl1* imprinted genes^37^. We cannot exclude that other genes could be affected by JWH-133 paternal exposure, predisposing offspring to metabolic and chronic diseases in adulthood.

More broadly, our findings support the notion that epigenetic marks can be modified by cannabinoid exposure and are in agreement with recent data reporting altered DNA methylation linked to *Cannabis *exposure in humans and animal models, in immune cells, in the brain and in sperm^13,38–40^. More importantly, this study highlights the role of CB_2_ overactivation in causing alterations of sperm DNA methylation that are inherited by the next generation with negative implications for offspring growth. However, we cannot exclude that reduced fetal growth may have adverse consequences on lifelong health^41^. Further studies will be required to understand if paternal exposure to cannabinoid could generate heritable epigenetic changes that would impact the fate of the next generations.

We further report that CB_2_ overactivation on the immune system, where CB_2_ is particularly abundant, affected hematological parameters inducing a significant decrease in the number of circulating lymphocytes. We believe that such effect in healthy males is probably mediated by the CB_2_ pathway as it has been previously reported in autoimmune disease models, in which activation of CB_2_ has been shown to suppress inflammation^42–45^.

In the context of *Cannabis* abuse, THC is believed to exert the majority of its actions as a partial agonist at both CB_1_ and CB_2_ receptors. However, it should be underlined that, besides THC, marijuana contains more than 100 other cannabinoids, whose mechanisms of action are still poorly known^46^. Thus it is possible that, some of them could act as full agonists of CB_2_, like JWH-133, or might modulate the effects of THC.

In conclusion, our findings highlight the potential consequences that paternal *Cannabis* exposure may have on immune function and reproductive success.

## Methods

### Animal care and ethics statement

All animal breeding, maintenance and research protocols were conducted as described in the project approved by the Ethics Committee of the Interdepartmental Service Centre - Station for Animal Technology (STA)-University of Rome Tor Vergata and in accordance with national and international laws and policies (Directive 2010/63/EU of the European Parliament and of the Council, Italian Legislative Decree 26/2014). In this study, we used CD-1 mice provided by the STA. Mice were randomly assigned and housed in standard clear plastic cages, kept in light/dark cycle of 12:12 hr and ventilation 10–20 times/h, with ad libitum water and food. Mice were kept in social groups at a constant temperature of 20 ± 2 °C, and relative humidity of 50 ± 10%. A block randomization method was used to randomize subjects into groups resulting in equal sample sizes. The investigators were blinded to the treatments.

### *In vivo* experimental procedure

P7 CD-1 male mice (n = 12) were intraperitoneally injected with JWH-133 (1.5 mg·kg^−1^) (Tocris, Bioscience, Bristol UK) for 5 consecutive days at 24 h intervals, followed by 2 days of rest. This procedure was repeated for five consecutive weeks. Control group (n = 12) was injected with the vehicle phosphate-buffered saline (PBS). The dosage of administrations were based on previous studies^10,14,15,26,45^. At the end of treatment, control (n = 6/12) and JWH-133-treated mice (n = 6/12) were randomly selected and crossed with untreated and sexually mature CD-1 female (single male pared with single female, n = 3 total females for each male) in order to analyze fertility and offspring health. Pregnancy was validated by visualizing the vaginal plug. Placentas and embryos from the two groups (control and JWH-133 treated) were recovered at E13.5 and E18.5 and morphologically analyzed. For each group, we recorded the number and weight of pups at delivery; weight of pups was recorded up to one month. For each group the number of animals analyzed was: n=80 embryos at E13.5, n=80 at E18.5 and n=80 newborns. Pups were generated from three independent treatments of male mice.

### Sperm analysis

At the end of treatment, control (n = 6/12) and JWH-133-treated mice (n = 6/12) were randomly selected and sacrificed to analyze sperm number, morphology and motility. Epididymes were removed and cauda were isolated for spermatozoa collection. Tissues (cauda) were immersed in PBS and needled to allow spermatozoa “swim-out”. Then spermatozoa samples were filtered, and sperm suspensions were analyzed to evaluate the morphology and the number of viable and motile cells under a light microscope, by using a Burker Chamber. Sperm motility was determined by count of duplicate measures of 200 sperms. This procedure was validated using double-blind test.

### Quantitative reverse transcription PCR (RT-qPCR)

Total RNA was isolated with TRI reagent (Ambion, Waltham, MA USA) according to manufacturer’s instructions by adding 20 µg of glycogen as a RNA carrier. Equal amounts of RNA were used to produce cDNA by means of PrimeScript RT Reagent Kit (Perfect Real Time) (Takara). Real-Time PCR reactions were performed by SYBR Premix Ex Taq II (Tli RNase H Plus) (Takara) with the following primers: Dnmt1(Forw: CTTCACCTAGTTCCGTGGCTA, Rev: CCCTCTTCCGACTCTTCCTT); Dnmt3a (Forw: GCACCAGGGAAAGATCATGT, Rev: CAATGGAGAGGTCATTGCAG); Dnmt3b (Forw: GGATGTTCGAGAATGTTGTGG, Rev: GTGAGCAGCAGACACCTTGA); Dnmt3l (Forw: CTGCTGACTGAGGATGACCA, Rev: GCTTGCTCCTGCTTCTGACT); Tet1 (Forw: TTTGGTTCGTGAGCGTGTAG, Rev: TGCAGGTACGCTTTTTGTTG); Tet2 (Forw: TGTTGTTGTCAGGGTGAGAATC, Rev: TCTTGCTTCTGGCAAACTTAC); Tet3 (Forw: AAGATAACAATCACGGCGTTC, Rev: CCGGATTGAGAAGGTCATCTAC); b-actin (Forw: CACACCCGCCACCAGTTCGC, Rev: TTGCACATGCCGGAGCCGTT).

### Methylated and hydroxymethylated DNA immunoprecipitation (MeDIP/hMeDIP) from sperm and placenta

Total DNA was isolated with TRI reagent (Ambion, Waltham, MA USA) according to manufacturer’s instructions and adding 20 µg of glycogen as DNA carrier. DNA immunoprecipitation was performed as previously described with some modifications^47^. Equal amounts of purified DNA from sperm samples (n = 6 for each group) or from E13.5 placentas (n = 6 for each group), were sonicated (40% amplitude; 0.5 cycle) to obtain fragments about 500 bp-300bp. Samples were heat-denatured for 10 min at 95 °C and immediately cooled on ice for 10 min. Sonicated DNA (2.5 µg) was diluted in IP buffer (10 mM Na-Phosphate buffer pH 7.0, 0.14 M NaCl, 0.05% Triton X-100) and pooled DNA was used for immunoprecipitation with 1 μg of anti-5mC (EpiGentek) or anti-5hmC (Active Motif) antibodies and normal rabbit/mouse IgGs as control. DNA–antibody mixtures were incubated 16 h and then 45 μl of the Protein-A or G Agarose beads (Millipore) were added and incubated for 2 h on a rotating platform at 4 °C. Beads and immunocomplexes were washed with IP buffer, digested with proteinase K (Sigma-Aldrich) and subjected to standard phenol–chloroform, ethanol precipitation procedure for DNA extraction. Air-dried DNA pellets were used for qPCR amplification. The following primers pairs were used for PCR analyses: *Plagl1* ICR (Forw: TAAGTAGTGACAACCGGGGC; Rev: TTTTGCTGCATCTCTGGCTG); *Peg10* ICR (Forw: CGCTTCAGCGTACGAACGAGCA; Rev: GTGCCGCAGTTTGTAGCGCATT); *H19* ICR (Forw: TGGCTCACTATAGGAAGGCAT; Rev: AAGTTGGCAGCATTTGGG).

### Hemocytometric analysis

Blood samples from JWH-133 treated and control adult mice (n = 10 for each group) were collected through a withdrawal from the orbital sinus. Approximately 100 μl of blood were drawn from each pup. Complete blood counts were obtained on an automated cell counter (Drew-3 Hematologic System).

### Flow cytometry

Splenocytes from control (n = 5) and JWH-133-treated (n = 5) mice were harvested and smashed through a 70 μm cell strainer (BD Falcon). After brief centrifugation, the cellular pellet was resuspended and incubated for 5 min in ACK lysing buffer (Lonza) in order to remove red blood cells. After extensive washing cells were counted and adjusted to a concentration of 1 × 10^6^ cells/mL in ice-cold FACS buffer (PBS, 0.5% BSA, 0.01% Sodium Azide). Cells were stained with a mix of monoclonal antibodies containing PE-Cy5 conjugated anti-CD3 (BD Pharmingen, clone 17A2), FITC conjugated anti-mouse B220 (BD Pharmingen, clone RA3-6B2), and PE-conjugated anti-mouse NK (BD Pharmingen, clone DX5), all diluted 1:100 in FACS buffer; after an incubation of 30 min at 4 °C, in the dark, cells were washed and fixed with 1% paraformaldehyde. Cells were then acquired and analyzed with a flow cytometer (model FACSCalibur BD) using ProCellQuest software (BD). At least 10.000 events for each sample were acquired.

### Histological analysis

Organs were fixed in Bouin’s solution or 4% paraformaldehyde, embedded in paraplast, sectioned at 5μm Leica-RM 2035 Microtome and stained with hematoxylin and eosin (H&E). Histological sections of placentas at E18.5 (n = 6 from each group) were stained with Periodic Acid Schiff (PAS) to visualize the spongiotrophoblast. Placental layers (labyrinth and spongiotrophoblast) were outlined manually and their areas were measured on five sections per placenta by using ImageJ. Histological sections of embryos at E13.5 and E18.5 were stained with H&E.

### Statistical analysis

Student’s t-test was used to test for differences between two independent groups. All statistical tests were carried out using the GraphPad Prism statistical analysis software package, version 6.0.

## Supplementary information

Supplementary information
