# Supplementary material for: Paternal activation of CB2 cannabinoid receptor impairs placental and embryonic growth via an epigenetic mechanism
Source: Sci Rep. 2019 Nov 19;9:17034. doi: 10.1038/s41598-019-53579-3 (PMC6863860; doi:10.1038/s41598-019-53579-3)

Paternal activation of CB_2 cannabinoid receptor impairs placental and embryonic growth via an epigenetic mechanism.

Elisa Innocenzi[1§], Emanuela De Domenico[1§], Fabio Ciccarone[2], Michele Zampieri[3], Gabriele Rossi[1], Rosella Cicconi[4], Roberta Bernardini[4], Maurizio Mattei[4,5] and Paola Grimaldi [1]*

Figure S1

Organ size and reproductive functions of F1 offspring.

A) JWH-133 treated males were crossed with mature unexposed females and organ weight of F1 offspring at P7 was analyzed. Organ weight of F1 pups from control and JWH-133 fathers at P7 are reported. B) Histogram reports the mean of the organ weight /body weight (n=9). (Liver= 25.89 ± 4.68 mg in JWH-133 vs 25.67 ± 6.25 mg in CTRL; Heart= 5.33 ± 0.9042 mg in JWH-133 vs 4.81 ± 1.16 mg in CTRL; Spleen= 4.52 ± 1.03 mg in JWH-133 vs 4.2 ± 1.263 mg in CTRL; Kidney= 5.6 ± 0.35 mg in JWH-133 vs 5.67 ± 0.55 mg in CTRL; Testis=0.915± 0.28 mg in JWH-133 vs 0.935 ± 0.20 mg in CTRL C) Histology of testis (Scale bar: 200 µm) and D) ovary sections (Scale bar: 75 µm) of F1 pups from CTRL or from JWH133 father stained with H&E. High magnification of gonads is shown. Scale bar: 75 µm. No abnormalities in the seminiferous tubules that contain spermatogonia (Sg) and Sertoli cells (SC) or in the ovary, in primordial (Pr) and primary (Pry) follicles, were detected. E) F1 males from control (n= 5) and JWH133 father (n=5) were crossed at adult age with sexually mature female mice never exposed to the drug. Histograms report mating rate, fertility rate and number of pups/litter of F1. Error bars represent SD.

A

Organ weights of F1 pups at P7

	CTRL	JWH133
Liver	0.163 ± 0.041	0.104 ± 0.017
Heart	0.031 ± 0.007	0.025 ± 0.005
Spleen	0.027 ± 0.008	0.018 ± 0.005
Kidney	0.036 ± 0.004	0.023 ± 0.003
Testis	0.006 ± 0.001	0.004 ± 0.001

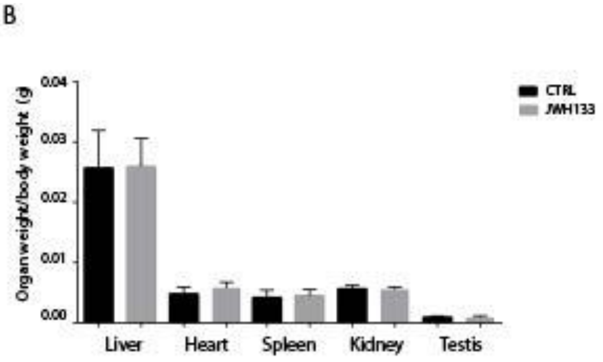

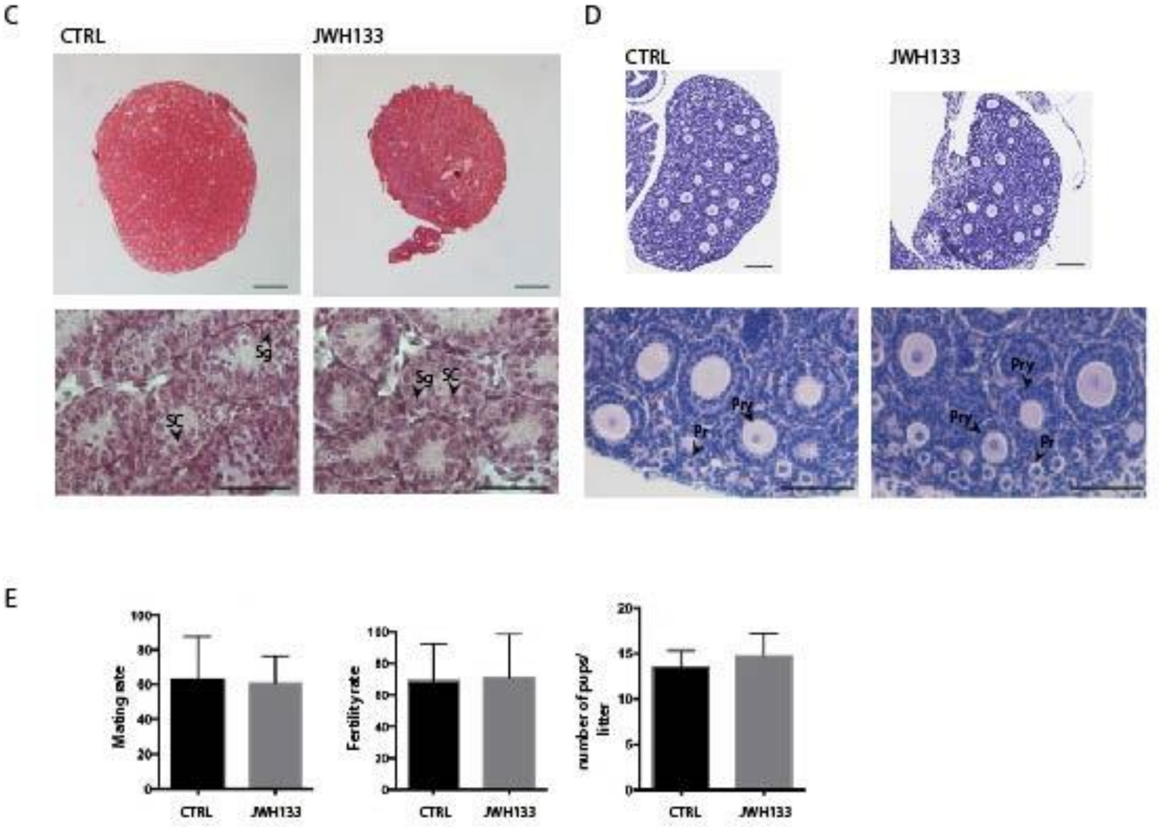

Supplement: Supplementary file 1 — Supplementary information [file 41598_2019_53579_MOESM1_ESM.pdf]
